# Correlation between Scratch Behavior and Tensile Properties in Injection Molded and Extruded Polymers

**DOI:** 10.3390/polym14051016

**Published:** 2022-03-03

**Authors:** Jasmina Germann, Timo Bensing, Martin Moneke

**Affiliations:** Darmstadt Institute of Plastics Engineering, Darmstadt University of Applied Sciences, Haardtring 100, 64295 Darmstadt, Germany; jasmina.germann@stud.h-da.de (J.G.); timo.bensing@h-da.de (T.B.)

**Keywords:** polymers, scratch testing, surface analysis, tensile properties, extrusion, microscopy

## Abstract

This study investigates the validity and applicability of the correlation between scratch and tensile properties for extruded polymer strands. The mechanical properties could be predicted for extruded samples, which allows skipping the step of injection molding and therefore enables a faster material development. Extruded polymer strands and tensile test specimens out of PMMA, PS, POM, PP and PE have been investigated. A correlation of the Young’s modulus and the elastic deformation as well as a correlation of the yield stress and the plastic deformation during scratching is given for both flat molded and cylindrical extruded specimens. SEM images of the scratch grooves are used to analyze the scratch deformation mechanism. The deformation mechanism correlates well to the variation coefficient of the indentation depth. Polarized light microscopy of thin cross sections of both types of specimens provides information about skin layer thickness and morphology. However, the optical analysis could not provide an explanation for the different levels of the indentation depth in the two specimen types. Further investigations should include a study of differences in process induced morphology and the effect of two layers with different mechanical properties, i.e., skin and center, on the stress and strain fields underneath the scratch.

## 1. Introduction

Plastics, especially engineering thermoplastics, need to be mechanically characterized since product designers use material properties such as Young’s modulus, tensile strength and others for choosing appropriate plastics, for first design steps and for structural simulations. Typically, a new plastic material will be compounded, injection molded into tensile test specimens and then tested to yield these material properties. Shortening this process by testing extruded strands gained from compounding could pose a huge benefit. If a correlation between the scratch behavior of extrudates and the Young’s modulus of tensile test specimens could be established, the material behavior can be predicted without the need for injection molding. Consequently, a faster development of new materials becomes possible and less material may be used.

Because of the great importance of a high scratch hardness of plastics, several studies concentrated on this topic in the past for almost three decades. Through an early study in 1966, it is known that the scratching process can be separated into an indentation and a sliding process [1]. The indentation process results in a normal pressure on the surface, while the sliding process results in shear strain, especially below the surface being just scratched. After this fundamental study, several researchers concentrated on the comparison of indentation and scratching of polymers [2,3].

The term scratch hardness was introduced by Briscoe et al. [3], who defined it as the ratio of the exerted load to the contact area. They also revealed a correlation of the indentation and scratch hardness for Poly(methyl methacrylate), when the test conditions are the same. Kurkcu et al. [4] focused on the scratch hardness of several plastics. In their constant load scratch test, polyethylene and polybutylene showed the lowest scratch hardness, whereas polycarbonate and poly(methyl methacrylate), melamine formaldehyde and styrene-acrylonitrile copolymer possessed the highest levels of scratch hardness. Besides the material itself, the scratch behavior of polymers depends significantly on the indenter geometry, normal load and sliding velocity, besides other influencing factors during scratching [4,5]. For example, a cone angle of the scratching indenter bigger than 150 degrees will lead to an elastic reaction or an ironing of the surface, while scratching with an indenter with a cone angle up to 90 degrees provokes a brittle or ductile machining as well as cracking [5]. In addition, a lower tip radius of the indenter tends to a higher scratch depth [1]. Gauthier et al. [6] further investigated the influence of the temperature and the tip velocity on the scratch behavior of PMMA. Increasing temperature and decreasing velocity lead to higher remaining scratch groove depth and width as well as to lower scratch hardness. Furthermore, our working group found that the position of the scratch on injection molded specimens has an influence on the resulting scratch hardness because of the molecular orientation of the polymer chains [7]. The scratch behavior can also be altered by adding fillers to the polymer. Molero et al. [8] examined PMMA with additional polyrotaxane. For one specific type of polyrotaxane, the required load for the onset of cracking was substantially higher than for neat PMMA. Petchwattana et al. [9] further examined the scratch hardness of PP depending on the amount of added calcium silicate. In comparison to unfilled PP, the scratch hardness depends on the applied load as well as the amount of the filler. For low normal loads, they found a significant increase of the scratch hardness with 10 % calcium silicate but a decrease with higher weight percentages.

Since scratches influence the optical material properties, some researchers focused on the visibility of scratches. Jiang et al. [10] evaluated the onset of scratch visibility depending on the scanning deformation and indenter angle. Briscoe et al. [11] additionally focused on the scratch visibility, with an emphasis on the difference of brittle and ductile thermoplastics. They found that the visibility of ironing or ploughing depends on the conditions during scratching as well as the material itself. In general, ductile plastics tend to a regular appearance of the scratch groove, whereas brittle plastics often show an irregular scratch groove with chip formation [12]. Zhang et al. [13] compared the critical normal load for the onset of scratch visibility to parameters obtained from FE-simulations. They found a strong negative correlation between the critical normal load and the tangential force during scratching as well as a good correlation between the critical normal load and the residual scratch depth. For measuring the scratch visibility, Briscoe et al. [14] used optical reflectivity. When the surface tends to a brittle behavior on scratching, the optical reflectivity is lower than for a surface, which shows plastic deformation.

The correlation between scratch and tensile properties could be shown by several studies in the past. Xiang et al. [1] linked a lower Young’s modulus to a higher scratch depth for a large number of polymers and the contact radius of the indenter. They also established the fact that a higher yield stress is accompanied by a lower tendency to plastic flow during scratching. Kurkcu et al. [4] also focused on the yield stress. They investigated the influence of rate-dependent mechanical properties on the scratch properties of flat polymer specimens, and found that the scratch process is mainly influenced by the tensile properties and especially the yield stress. Using FE-simulations, Zhang et al. [13] found that the residual scratch depth is significantly influenced by the yield stress. With increasing yield stress, the residual scratch depth decreases. Another study observed the influence of blend compositions on the correlation of scratch and material properties [15]. With a higher content of TPU, the blend of TPU/PMMA shows a higher indentation depth, elongation at break and Charpy impact strength. In contrast, the tensile modulus, tensile strength and Shore D hardness are negatively correlated to the content of TPU.

As described, several studies revealed a connection between tensile and scratch properties. As a result, a correlation model between these properties can be used to predict the tensile properties of a polymer by just performing a single scratch test. It would allow for accelerating a material development process because of the necessity for molding tensile test specimens and carrying out standard tensile tests ceasing. The current study focuses on extruded polymer strands, which are a product of the compounding process itself. Therefore, the study has the aim to investigate the relation between the scratch properties of cylindrical, extruded polymer strands and the tensile properties of tensile test specimens. To this end, three ductile (PE, PP, POM) and two brittle (PS, PMMA) thermoplastic polymers are scratched. Tensile tests are performed to determine mechanical properties like the Young’s modulus. The last step is the comparison of the resulting test data to develop a correlation model. Since the examination is performed on two different types of specimens, further influence factors are identified on the basis of microscopic analysis.

## 2. Materials and Methods

### 2.1. Materials

For this work, several ductile and brittle thermoplastics have been chosen. All of these thermoplastics are used in applications where a high scratch resistance is required, e.g., in coatings or in automotive interior. Furthermore, the chosen thermoplastics cover a large range of mechanical properties, which is needed for the development of a correlation model. Poly(methyl methacrylate) (PMMA) and polystyrene (PS) were picked as amorphous polymers with a brittle behavior in mechanical tests, whereas polyoxymethylene (POM), polypropylene (PP) and polyethylene (PE) are semi-crystalline polymers with a ductile behavior. The specific trade names are Hostalen 5231H (PE), Plexiglas^®^ 6N (PMMA), Hostaform^®^ C 9021 (POM), Moplen HP501H (PP) and Styrolution^®^ PS 158N. The materials do not contain any additional fillers or colorants. All thermoplastics were obtained as granules. For the experiments, two types of specimens were necessary. On the one hand, extruded polymer strands were produced with a twin-screw extruder. The extruded polymer strands of all five types of thermoplastics reveal almost the same diameter along the strand length. On average, they had a diameter from 2.5 to 3.5 mm. On the other hand, tensile test specimens of type 1A according to ISO 527-2 were produced. Both strands and tensile test specimens were stored in airtight bags till testing. Before testing, strands and tensile test bars were conditioned at (23 ± 2) °C and (50 ± 10)% relative humidity for 88 h following ISO 291. The same atmosphere was maintained during tensile and scratch testing to avoid an influence by environmental conditions on the test results.

### 2.2. Tensile Tests

Tensile tests are done according to ISO 527-2 on a universal testing machine type Z050 by ZwickRoell GmbH & Co. KG, Ulm, Germany. According to ISO 527-1, the traverse velocity is 1 mm/min for determining Young’s modulus and 50 mm/min for higher elongations. The mechanical properties used for correlation are the Young’s modulus and the yield stress. For each material, 5 specimens are tested. From the results, the mean value and the standard deviation are calculated.

### 2.3. Scratch Tests

Scratch tests were conducted with the Universal Surface Tester 1000 (UST 1000) from Innowep GmbH, Würzburg, Germany. The scratch method employed here comprises three steps. At first, the height profile along a straight line is measured by moving the indenter over the surface at a constant velocity and with a normal load of 10 mN to ensure contact. After being retracted to the starting position, in the second step, the indenter moves along the same line with the same velocity as in the first step but with a higher normal load up to 1000 mN. In the second step, a scratch is produced, the depth (height profile) of it being measured while scratching. In a third step, again, the surface topography is scanned along the same line, which now is a scratch, with the same parameters as in step 1. The UST 1000 can apply normal loads between 10 and 1000 mN and scratch velocities up to 2.5 mm/s. The results of the scratch test are the elastic and plastic deformation as well as the total deformation, all being differences between height profiles measured in the three steps described before. The elastic deformation is calculated from the height profile measured during the third step of the scratch test minus the height profile measured during step 2 (scratching). The plastic deformation results from the height profile in step 1 minus the height profile in step 3. The total deformation corresponds to the sum of elastic and plastic deformation or the difference between the height profiles from step 1 minus step 2, respectively.

**Figure 1:**
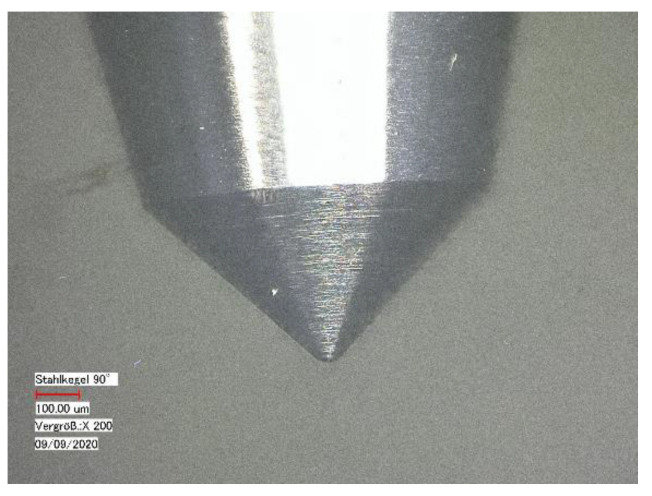
Light micrograph of the used steel indenter with a cone angle of 90° and a tip radius of 40 μm, magnification: 200×.

In the present study, a scratch was created on both tensile test specimens and extruded polymer strands. The indenter used is a steel indenter with a cone angle of 90° and a tip radius of 40 μm (Figure 1).

**Figure 2:**
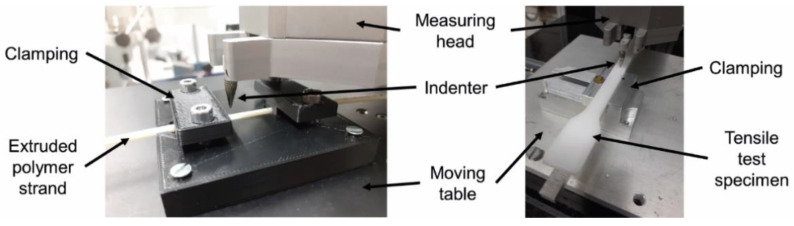
View on the scratch tester UST 1000; (**left**) clamping for extruded polymer strands; (**right**) clamping for tensile test specimens.

The length of the scratch is 5 mm. To investigate the influence of the scratch load and velocity, these parameters are varied. While the load for measuring the surface topography constantly is set at 10 mN, scratch loads are 300, 600 and 900 mN, respectively. Scratch velocities are 0.5, 1.0 and 1.5 mm/s, respectively. Every combination of thermoplastic, load and velocity is repeated five times for statistical purposes with scratches being in close distance to each other to exclude the influence of changing molecular orientation or degree of crystallinity along the flow path in tensile test specimens. To fix the used extruded polymer strands as well as the tensile test specimens for scratching, clampings have been developed (Figure 2). The extruded polymer strands are fixed by two hold-down clamps in a v-nut, whereas tensile test specimens are mounted in a vice-like clamping.

### 2.4. Microscopy

Microscopy has been used for the characterization of both the morphology in test specimens and the scratch grooves.

Polarized light microscopy in transmission was performed on unscratched specimens to investigate the influence of the morphology on scratch properties. For this, sections were cut from specimens transversal to the flow direction during processing with a microtome and primed on specimen slides. For optical microscopy, a Keyence VHX 500 with a VH-Z100R lens was used.

After scratch testing, the scratch groove was characterized by scanning electron microscopy (SEM). For this, the specimens were cut to pieces of a few millimeters length and coated with a gold layer. The SEM is a Hitachi SU5000 from Hitachi High-Tech Corporation, Tokyo, Japan.

## 3. Results and Discussion

### 3.1. Scratch Testing

**Figure 3:**
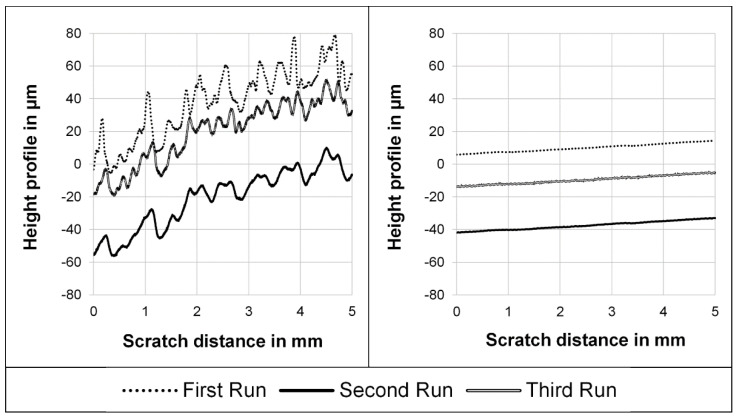
Height profiles of a scratch on an extruded polymer strand surface (**left**) in comparison to a tensile test specimen surface (**right**); material: PE, scratch velocity: 0.5 mm/s, indentation force: 900 mN.

In Figure 3, an example of a scratch result by the UST 1000 is given. As mentioned before, the indenter moves over the surface three times, named first run, second run and third run in Figure 3. The left graph shows the height profiles for the three runs on the surface of an extruded polymer strand from PE, while the right graph is for a tensile test specimen from PE employing the same scratch parameters. For the extruded polymer strand of PE, a rough surface is detected from the irregular height profile of the first run. The height profile of the tensile test specimen is quite straight over the scratch length during the first run. Thus, the tensile test specimen surface appears to be smoother than the one of the extruded polymer strand. Compared to the scratch results of the other thermoplastics, it becomes obvious that the differences in surface topography of both types of specimens are not generally valid for all tested thermoplastics. However, since only the deformation quantities resulting from the difference of the height profiles are evaluated, the measurement results of all specimens are comparable despite different surface topographies.

In general, the average total deformation on extruded polymer strands is higher than on tensile test specimens. The fact that the elastic deformation is a bit more than twice as high as the plastic deformation is valid for both types of specimens.

### 3.2. Factors Influencing Scratch Test Results

**Figure 4:**
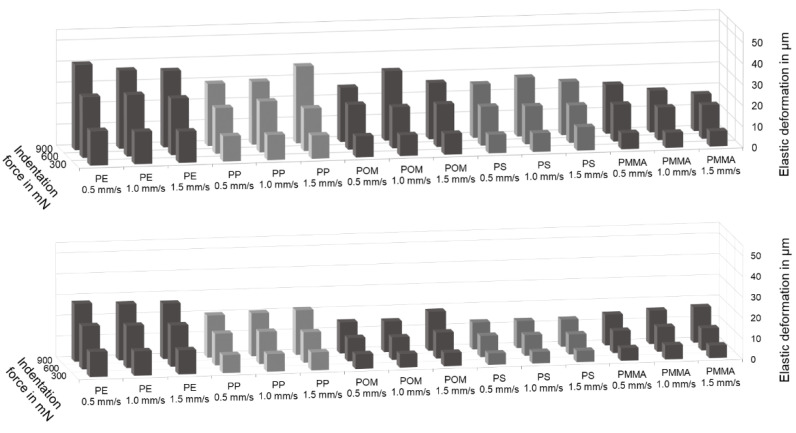
Elastic deformation as a function of indentation force and scratch velocity measured at extruded polymer strands (**top**) and tensile test specimens (**bottom**) of PE, PP, POM, PS and PMMA.

At first, some scratches have been performed with varying indentation force and scratch velocity on both types of specimens used. Based on this parameter study, the influence of the two parameters within the chosen variation range on the scratch results can be studied. The results are presented in Figure 4.

Figure 4 shows that the elastic deformation is dependent on the indentation force and the type of thermoplastic. For both types of specimens, the elastic deformation rises for every plastic with increasing indentation force. For each indentation force, the elastic deformation is distinctly different for all materials. The largest elastic deformation is measured for PE, the lowest for PMMA and PS. In contrast, the scratch velocity does not count as an influencing variable for the elastic deformation. In general, the elastic deformation does not vary more than ten percent for the different executed scratch velocities for every specific plastic at all indentation forces. The described relation between the scratch parameters and the deformation is also valid for the plastic deformation (not shown). Consequently, the following investigation is made with different forces and thermoplastics. Since the results for different velocities differ in no significant height, the indentation depths are averaged over all velocities for the correlation model development.

### 3.3. Correlation between Scratch and Tensile Properties

The material properties gained from tensile tests are listed in Table 1.

**Figure 5:**
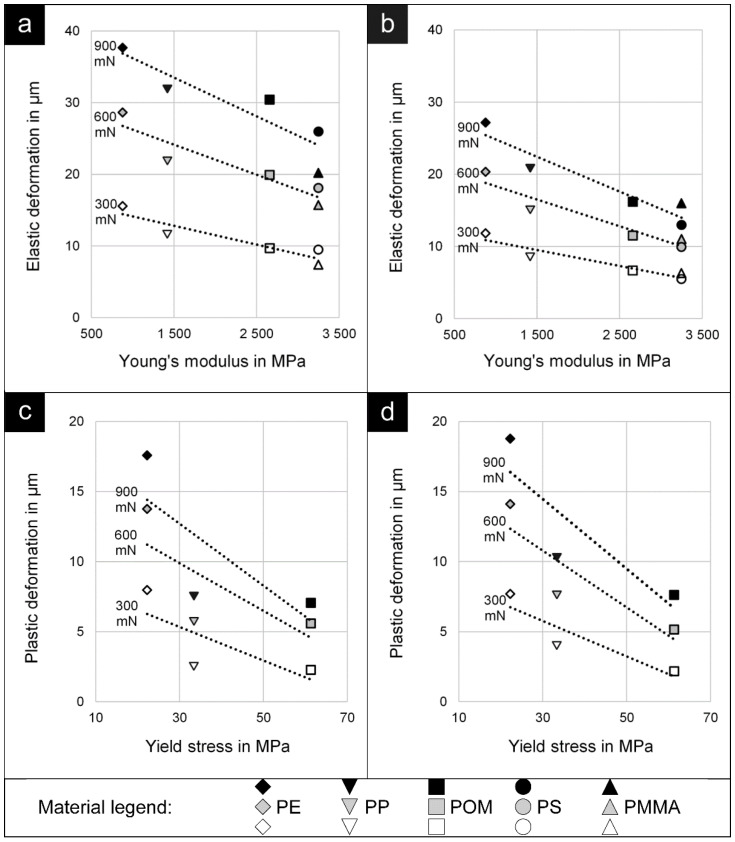
Linear regression of plastic deformation versus Young’s modulus for extruded polymer strands (**a**) and tensile test specimens (**b**) as well as linear regression of plastic deformation versus yield stress for extruded polymer strands (**c**) and tensile test specimens (**d**).

In a next step, the elastic deformation of all thermoplastics is compared to the Young’s modulus, while the plastic deformation of the ductile thermoplastics is compared to the yield stress (Figure 5). The tensile parameters, shown on the abscissa, were gained from tensile testing (Table 1). Deformation values come from scratch tests made on extruded polymer strands (Figure 5a,c) and on tensile test specimens (Figure 5b,d). Both the elastic and plastic deformation decrease with increasing tensile properties. In particular, these values are related linearly with a negative correlation coefficient in the considered range of the tensile properties. The relation is valid for both types of specimens, despite their different manufacturing. The only difference between the correlations is the position and the slope of the regression lines. Concerning the correlation of the elastic deformation and the Young’s modulus, the gradient is higher on extruded polymer strands. On the contrary, the gradient of the plastic deformation over the yield stress is higher on tensile test specimens.

### 3.4. Surface Deformation Caused by Scratching

**Figure 6:**
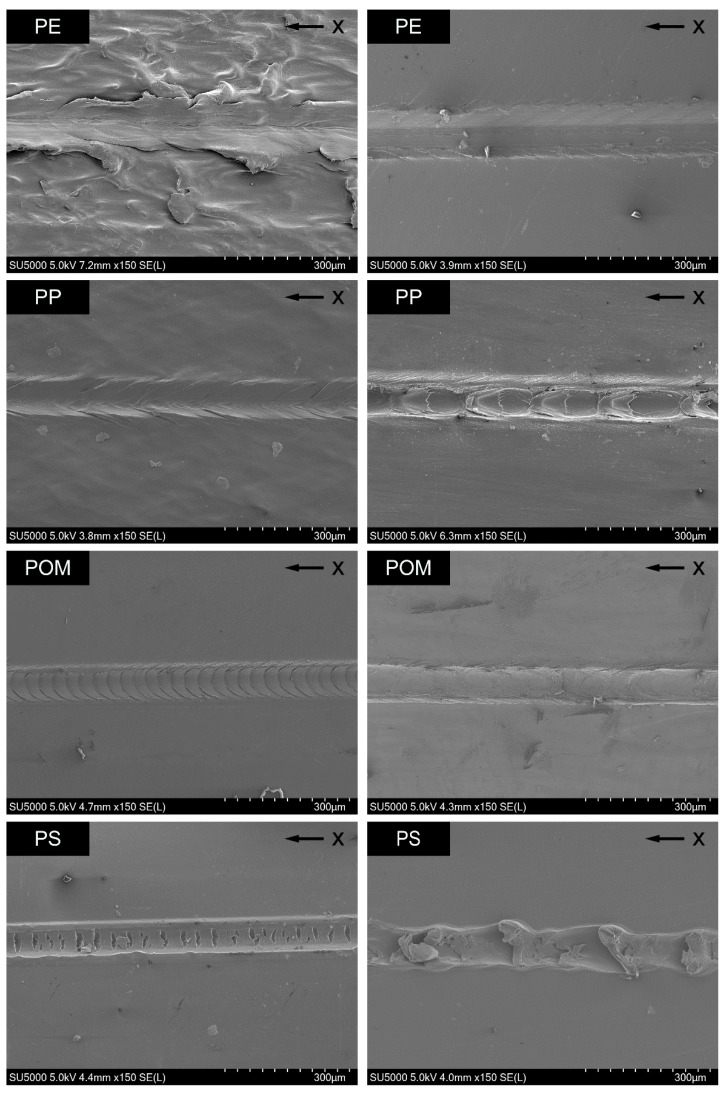

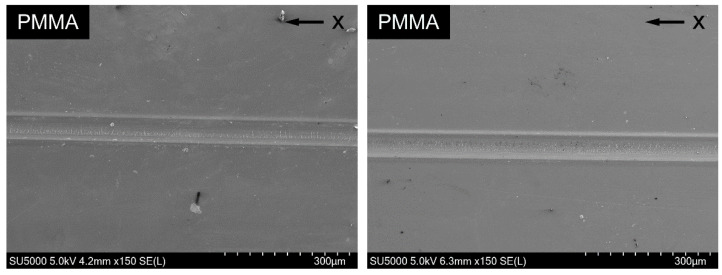
Scratch grooves of different polymers observed using SEM, scratch velocity: 0.5 mm/s, normal force: 900 mN; (**left**) extruded polymer strands, (**right**) tensile test specimens; “x” marks the scratch direction.

Since the scratch results yield different height profile patterns for the different materials and specimen types, the surface topographies and scratch grooves are analyzed with an SEM. Figure 6 shows a representative micrograph for every thermoplastic at the same velocity–force combination.

The extruded polymer strand of PE has a rough surface and a scratch edge with irregular waves. The scratch groove itself shows irregular patterns. In contrast, the surface of the tensile test specimen of PE is smooth, and the scratch groove is more regular with waves with low amplitude and higher frequency in comparison. Consequently, the indentation varies more for the extruded polymer strand of PE over the scratch length. The extruded polymer strand of PP possesses a slightly rough surface and the scratch exhibits regular waves, with the particularity that the waves seem to protrude beyond the edge of the scratch groove. In contrast, the scratch groove on the tensile test specimen of PP shows pronounced edges with no waves inside but a regular pattern of repeating oval areas with nearly the same dimensions. These areas are due to material accumulations during scratching. For POM, the scratch groove on the extruded polymer strand shows extremely regular and weak waves, but, on the tensile test specimen, it looks smoother and at the same time more irregular. In particular, the scratch edge is not as regular as the one on the extruded polymer strand. For PS, the scratch on the extruded polymer strand has a regular border with large perpendicular cracks in the scratch ground. The scratching on a tensile test specimen of PS leaves an irregular groove with regularly material accumulations. The scratch groove for both types of specimens of PMMA is smooth with a linear border. Only a few little cracks are spread over the scratch ground. Waves cannot be detected. The micrographs show that the scratch groove on both types of specimens of brittle plastics, i.e., PS and PMMA, does not contain any waves. Instead, cracks are common except on the groove on a tensile test specimen of PS.

From the comparison of the scratch profiles on specimens of PE and the scratch appearance seen by microscopy, it can be concluded that the scratch profiles reflect the scratch deformation mechanism. The irregular waves on the extruded polymer strand of PE result in a large variation of the indentation depth in the scratch profiles. Furthermore, the height profiles suggest that elevations on the rough surface are slightly pushed forward during scratching (see Figure 3). On the other hand, the regular scratch groove with weak waves on the tensile test specimen leads to a regular indentation depth profile.

**Figure 7:**
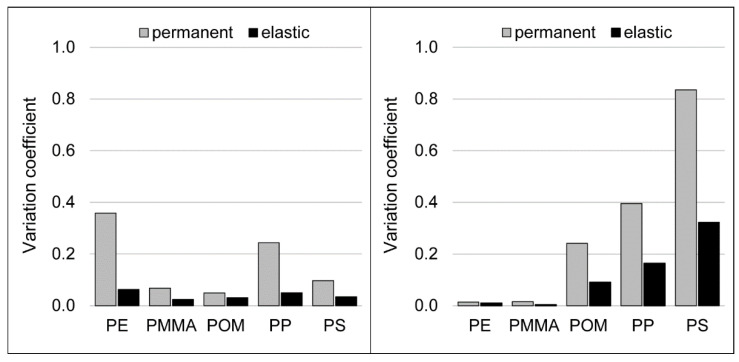
Variation coefficient of the indentation depth as an average over five scratches on extruded polymer strands (**left**) and tensile test specimens (**right**); scratch velocity: 0.5 mm/s, indentation force: 900 mN.

The comparison between scratch profile and scratch pattern seen by microscopy is done for all specimens. Since the deformation mechanism during scratching can be connected to the variation of the indentation, the variation coefficient, which is calculated as the standard deviation divided by the mean value of the indentation, can be used for the characterization (Figure 7).

For extruded polymer strands, PE and PP have the highest variation coefficient. These materials are the thermoplastics with the roughest surface and therefore have the most irregular topographies. For tensile test specimens, the maximum of variation coefficient can be seen for PS, with PP revealing the second highest value. These thermoplastics show the biggest amount of material accumulations during scratching.

### 3.5. Skin Layer Morphology

**Figure 8:**
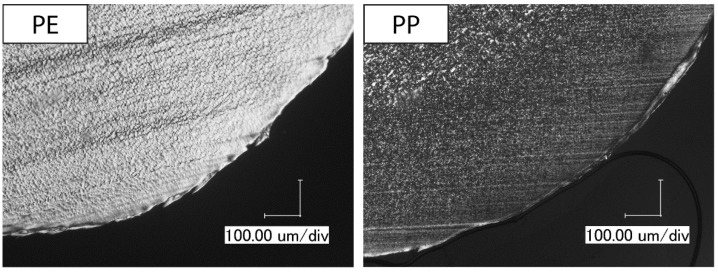

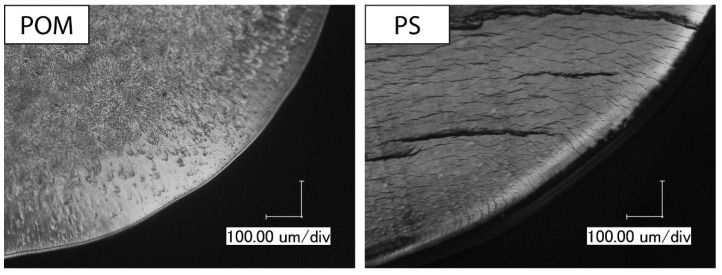
Skin layer morphology of extruded polymer strands observed using polarized light microscopy, magnification: 300×.

Since the scratch tests on the extruded polymer strands consistently show higher total indentation depths than on the tensile test specimens, thin cross sections of both types of specimens are analyzed with polarized light microscopy to gain information about their morphology. In particular, the skin layer thickness is established with the microscope by measuring from the surface of the samples to the onset of the uniform-appearing center. Due to the optical properties of PMMA, micrographs could not be generated. Figure 8 shows the micrographs of extruded polymer strands for the remaining four materials.

PE shows a regular morphology over its entire cross section with a small skin layer with a thickness between 38 and 68 µm. The other investigated polyolefin, PP, displays a skin layer with a thickness of around 370 to 452 µm, which consists of smaller crystalline structures than the center. POM has a comparable appearance with a skin layer thickness of 197 to 211 µm. Again, the center of the extruded polymer strand possesses larger crystallites than the skin layer. For PS, a skin layer cannot be identified. Some cracks can be seen in the PS strand, which are caused during sample preparation due to its brittle behavior.

**Figure 9:**
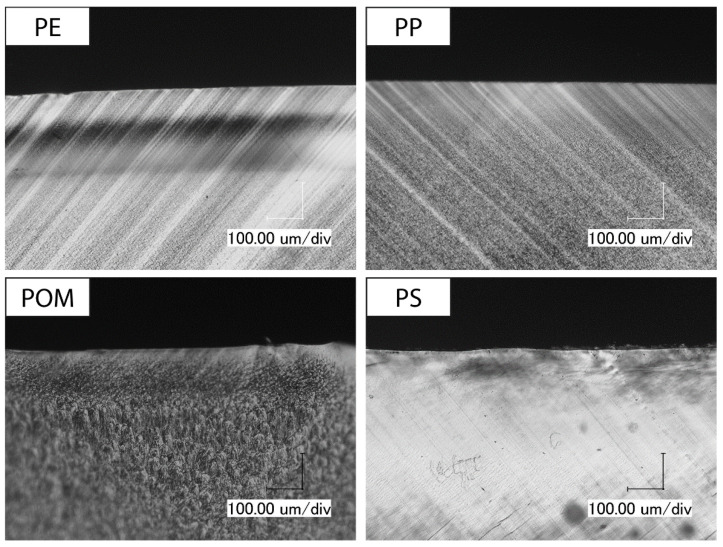
Skin layer morphology of tensile test specimens observed using polarized light microscopy, magnification: 300×.

Next, tensile test specimens are analyzed and compared to the extruded polymer strands. Figure 9 shows the micrographs of thin cross sections of the tensile test specimens.

Both PP and POM reveal a skin layer in tensile test specimens with smaller crystallites than in the center, just like in the extruded polymer strands. The skin layer of PP specimens has a thickness from 137 to 163 µm and of POM specimens from 140 to 162 µm. The skin layer in the tensile test specimen of PE has a thickness from 239 to 245 µm. In the cross section of PS, no skin layer can be detected.

The skin layer thickness for all specimens is summarized in Table 2. Additionally, the table contains the total deformation during scratching for a better comparison of both parameters.

For a given polymer, the total deformation increases with normal force as would be expected. In addition, for each polymer, the total deformation is smaller for the injection molded samples compared to the extruded strands for each of the three normal loads.

PE is the only polymer from the three semi-crystalline polymers where the total deformation is of the same order as the skin layer thickness, while, for POM and PP, the skin layer thickness is a minimum four times larger than the total deformation. In other words, the stress field underneath the scratch vanishes to zero within the skin layer for POM and PP, while, for extruded strands from PE, the stress field extends through the skin layer into the center.

The skin layer thickness itself is smaller for injection molded POM and PP compared to extruded strands, while it is the other way round for PE. Since, in injection molding, temperature gradients from center to skin are larger than in extrusion, one would expect a more pronounced skin layer in injection molded samples. In addition, higher shear rates which are common in injection molding would lead to a more pronounced skin layer. Therefore, the observed skin layer thickness does not fit expectations and is not suitable to explain the total deformation in the different polymers.

## 4. Conclusions

This study investigates the validity and applicability of the correlation between scratch and tensile properties known from injection molded samples for cylindrical, extruded polymer strands. For different indentation forces and different polymers, the correlation between the Young’s modulus and the elastic deformation on the one hand and the yield stress and the plastic deformation on the other hand for extruded and injection molded specimens can be derived from the experiments. The graphical comparison of the scratch and the tensile properties shows a different slope of their regression lines. The correlation between the elastic properties is stronger on extruded polymer strands, while the correlation of the plastic properties is stronger on tensile test specimens.

The scratch deformation mechanism in both types of specimens is determined via SEM. For all five thermoplastics, a strong correlation of the deformation mechanism with the variation coefficient of the indentation depth can be established. The influence of brittle and ductile thermoplastics behavior on scratch groove appearance can be seen.

Thin cross sections of all specimens were analyzed with polarized light microscopy in order to obtain information about the skin layer thickness and the morphology. From the micrographs, the differences between the two types of specimens, tensile test specimens and extruded strands, becomes obvious. Since molded specimens are subjected to higher shear and cooling rates, the morphology of their transverse sections is different to those of extruded polymer strands. However, the skin layer thickness itself does not correlate with the indentation depth. While POM and PP have a higher skin layer thickness in extruded strands, the opposite is true for PE. Therefore, further investigations are necessary to explain the influence of morphology on the indentation depth. Further studies could focus on the degree of crystallinity in the skin layer as one typical feature of process induced morphology and the effect of two layers with different mechanical properties, i.e., skin and center, on the stress and strain fields underneath the scratch and hence the indentation depth.

## Figures and Tables

**Table 1:** Tensile properties of the thermoplastics according to ISO 527 with conditioning according to ISO 291 (the standard error of the mean is given in parentheses).

Thermoplastic	Young’s Modulus in MPa	Yield Stress in MPa
PE	880 (13)	22.3 (0.5)
PMMA	3250 (6)	-
POM	2660 (10)	61.3 (0.1)
PP	1420 (9)	33.4 (0.1)
PS	3250 (15)	-

**Table 2:** Comparison of the average of the total deformation during scratching and skin layer thickness observed through light microscopy (the standard error of the mean is given in parentheses).

	Material	Total Deformation in µm at an Indentation Force of…			Skin Layer Thickness in µm
		300 mN	600 mN	900 mN	
Extruded polymer strands	PE	23.7 (0.5)	42.4 (0.6)	55.2 (0.9)	38…68
	POM	11.9 (0.4)	25.4 (0.9)	36.0 (1.0)	197…211
	PP	14.4 (0.4)	27.9 (1.2)	39.4 (0.8)	370…452
	PS	12.1 (0.6)	23.4 (0.9)	32.9 (1.1)	-
Tensile test specimens	PE	19.6 (0.2)	34.3 (0.2)	45.9 (0.3)	239…245
	POM	8.9 (0.4)	17.8 (1.1)	23.5 (1.0)	140…162
	PP	12.6 (0.2)	22.9 (0.4)	31.4 (0.7)	137…163
	PS	7.3 (0.2)	13.7 (0.2)	18.8 (0.3)	-

## Data Availability

Not applicable.
